# Identification of exon skipping events associated with Alzheimer’s disease in the human hippocampus

**DOI:** 10.1186/s12920-018-0453-8

**Published:** 2019-01-31

**Authors:** Seonggyun Han, Jason E. Miller, Seyoun Byun, Dokyoon Kim, Shannon L. Risacher, Andrew J. Saykin, Younghee Lee, Kwangsik Nho, for Alzheimer’s Disease Neuroimaging Initiative

**Affiliations:** 1 Department of Biomedical Informatics, University of Utah School of Medicine, Salt Lake City, UT USA; 2 Biomedical and Translational Informatics Institute, Geisinger Health System, Danville, PA USA; 3 The Huck Institutes of the Life Sciences, Pennsylvania State University, University Park, PA USA; 4 Center for Neuroimaging, Department of Radiology and Imaging Sciences and Indiana Alzheimer Disease Center, Indiana University School of Medicine, Indianapolis, IN USA; 5 Center for Computational Biology and Bioinformatics, Indiana University School of Medicine, Indianapolis, IN USA

**Keywords:** Alzheimer’s disease, Exon skipping, RNA-sequencing, RELN, NOS1, Neuroimaging, Human hippocampus

## Abstract

**Background:**

At least 90% of human genes are alternatively spliced. Alternative splicing has an important function regulating gene expression and miss-splicing can contribute to risk for human diseases, including Alzheimer’s disease (AD).

**Methods:**

We developed a splicing decision model as a molecular mechanism to identify functional exon skipping events and genetic variation affecting alternative splicing on a genome-wide scale by integrating genomics, transcriptomics, and neuroimaging data in a systems biology approach. In this study, we analyzed RNA-Seq data of hippocampus brain tissue from Alzheimer’s disease (AD; *n* = 24) and cognitively normal elderly controls (CN; *n* = 50) and identified three exon skipping events in two genes (*RELN* and *NOS1*) as significantly associated with AD (corrected *p*-value < 0.05 and fold change > 1.5). Next, we identified single-nucleotide polymorphisms (SNPs) affecting exon skipping events using the splicing decision model and then performed an association analysis of SNPs potentially affecting three exon skipping events with a global cortical measure of amyloid-β deposition measured by [^18^F] Florbetapir position emission tomography (PET) scan as an AD-related quantitative phenotype. A whole-brain voxel-based analysis was also performed.

**Results:**

Two exons in *RELN* and one exon in *NOS1* showed significantly lower expression levels in the AD participants compared to CN participants, suggesting that the exons tend to be skipped more in AD. We also showed the loss of the core protein structure due to the skipped exons using the protein 3D structure analysis. The targeted SNP-based association analysis identified one intronic SNP (rs362771) adjacent to the skipped exon 24 in *RELN* as significantly associated with cortical amyloid-β levels (corrected *p*-value < 0.05). This SNP is within the splicing regulatory element, i.e., intronic splicing enhancer. The minor allele of rs362771 conferred decreases in cortical amyloid-β levels in the right temporal and bilateral parietal lobes.

**Conclusions:**

Our results suggest that exon skipping events and splicing-affecting SNPs in the human hippocampus may contribute to AD pathogenesis. Integration of multiple omics and neuroimaging data provides insights into possible mechanisms underlying AD pathophysiology through exon skipping and may help identify novel therapeutic targets.

**Electronic supplementary material:**

The online version of this article (10.1186/s12920-018-0453-8) contains supplementary material, which is available to authorized users.

## Background

Alzheimer’s disease (AD) is a progressive neurodegenerative disorder pathologically characterized by an accumulation of both toxic amyloid-β plaques and neurofibrillary tau tangles in the brain [1]. Twin studies as well as more recent large-scale genome-wide association studies (GWAS) have demonstrated that genetic susceptibility factors play an important role in the development of the AD, although there is still a substantial portion of missing heritability to be identified [2, 3]. Increasing evidence suggests that widespread transcriptional changes accompany the onset and progression of AD [4–8]. In particular, the aberration in the control of gene expression by alternative splicing is implicated in AD [9–12]. Previous whole transcriptome sequencing analyses revealed gene expression and alternative splicing changes in the AD-affected brain regions [4, 10–12]. Several alternatively spliced AD candidate genes such as *CLU* and *CD33* were reported to be associated with AD pathogenesis [13, 14]. Thus, it could provide valuable information on the underlying pathology associated with the AD to identify other alternative spliced genes and AD-associated single-nucleotide polymorphisms (SNPs) affecting splicing regulation.

Alternative splicing is the process by which a single gene can produce multiple RNA isoforms through the splicing in and out of different portions of the transcript. Although it is an important mechanism for increasing biological complexity through generating tissue-specific transcript, miss-splicing can lead to different disease states. For example, in humans, more than 90% of genes are alternatively spliced [15] and generating 100, 000 proteins through different usage of exons (i.e. alternately spliced exons) [16]. Furthermore, such transcripts or certain exon skipping are expressed in the tissue- and disease-specific manner. Especially more genes are alternatively spliced in the brain than other tissues [16], and specific exons are brain-specially skipped or included in AD-associated genes including *APP* [13, 16], *PSEN1* [17], *PSEN2* [17], *APOE* [16], and *MAPT* [18–20].

SRE is an ancillary cis-acting element as a part of the splicing machinery that assists a spliceosome to correctly recognize the exon-intron boundary by recruiting activator or repressor Trans-acting RNA-binding proteins (RBP) [21]. There are four types of SREs, exonic splicing enhancers (ESEs), exonic splicing silencers (ESSs), intronic splicing enhancers (ISEs), and intronic splicing silencers (ISSs). Mutation in any sites of SREs changes the binding accuracy of spliceosome to the splice sites and potentially result in the aberrant exon skipping events producing disease-causing proteins. Furthermore, 15% of disease-causing mutation is estimated to be associated with splicing including SREs and splice sites [22–24], and we have previously demonstrated that alternative splicing is useful for identifying disease-associated variation in the human genome [25, 26]. It remains difficult to identify novel genes and molecular mechanisms associated with splicing that underlie AD pathological hallmarks due to the nature of studying the brain and neuropathological traits.

This study explores how transcriptomics, genomics and neuroimaging endo-phenotypes can be leveraged as a means to clarify our understanding of the genetic architecture of AD. Using RNA-Seq data from cognitively normal elderly controls and AD-affected human hippocampal tissues, alternative splicing isoforms were evaluated by measuring exon skipping. A computational pipeline to identify exon skipping events using RNA-Seq data and a splicing decision model to identify actionable loci among common SNPs for gene regulation were applied in this study to gain insights into the functionality of the variations and emphasized their importance for the AD pathology [27]. We identified SNPs affecting exon skipping by analyzing sequence-driven alternative splicing models and by scanning the genome for the regions with putative splicing regulatory elements (SREs) motifs [26, 27]. Aberrant alternative splicing sites were detected that associate with the AD using SNPs within regions affecting the exon skipping as associated with AD-related neuroimaging biomarkers, a global cortical measure of amyloid-β deposition measured by [^18^F] Florbetapir position emission tomography (PET) scans. These results provide a new link between alternative splicing changes and AD.

## Methods

### Study sample and RNA-sequencing data analysis

RNA-Seq data (bam files) were downloaded from the Allen Brain Atlas (http://human.brain-map.org/). RNA was isolated from the hippocampus tissue of brains of AD patients (AD; *n* = 24) and non-AD elderly controls (CN; *n* = 50) from the Adult Changes in Thought (ACT) study. The ACT study is a longitudinal population-based prospective cohort study of brain aging and incident dementia in the Seattle metropolitan area, as described in detail in previous studies [28, 29]. RNA sequencing was performed using an Illumina HiSeq 2500 with v4 chemistry, producing a minimum of 30 M 50 bp paired-end clusters per sample. Raw read files (bam files) were aligned to the GRCh38 reference genome, as described in detail (http://aging.brain-map.org/). The average reads of all individual participants (*n* = 74) were 55,015,989.

### Identification of exon skipping events

**Fig. 1:**
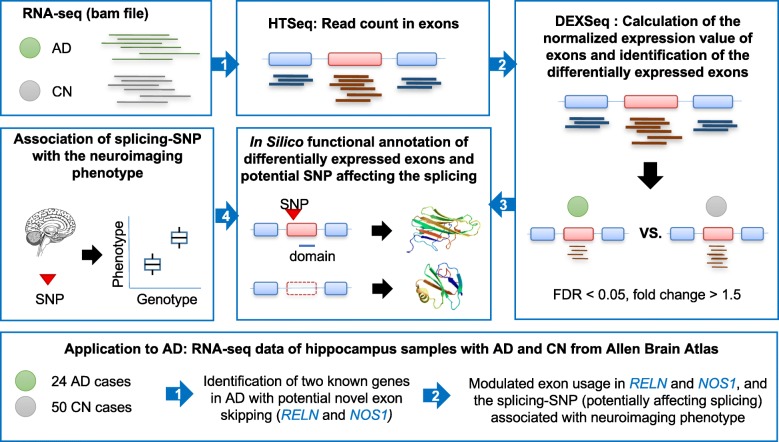
Computational pipeline for identifying AD-associated exon skipping events and SNPs affecting exon skipping

A bioinformatics splicing decision model based on our previous study [27] was implemented to identify exon skipping events and genetic variation affecting exon skipping using RNA-Seq data on a genome-wide scale in the human hippocampus (Fig. 1). First, to estimate the normalized usage for each exon, total reads for each exon were counted from RNA-seq data (bam files with the mapped reads) using HTSeq [30, 31] and there were only exon reads, but no junction reads. Junction reads are crucial information to know which exon structure is changed. However, as we obtained the exon reads from the bam files, we run DEXSeq to measure exon expression level that basically uses exon reads only [31]. Next, expressed exons (exon skipping events) between AD and CN were identified using a generalized linear regression model in DEXSeq. The statistical cutoff as a significantly differentially expressed exon (exon skipping) was false discovery rate (FDR)-corrected *p*-value < 0.05 and fold change > 1.5, which was calculated with p.adjust function in R, i.e., correction for multiple comparisons was performed using the FDR-based multiple comparison adjustment with the Benjamini-Hochberg procedure at a 0.05 level of significance.

### Identification of SNPs in splicing regulatory elements associated with exon skipping events

We have developed a splicing decision model for identifying SNPs affecting splicing regulatory elements (SREs) with exon skipping by using alignment information for four alternative splicing datasets from the UCSC genome browser: mRNAs from GenBank [32], Ensembl Gene Predictions [33], AceView Gene Models [34], and UCSC known genes [35] and a set of predicted hexameric SRE motifs, as described in detail in previous publications [26, 27]. We searched for all potential SRE sites that are perfectly matched with any of these hexamers in intragenic regions (exons and introns). Our study included three types of SREs available for this time, ESE, ESS, and ISE according to its location and function without ISS as the data of ISS hexameric sequences are not available. We then compiled genotype data and SRE regions with skipping of the adjacent exon to intron or skipping of the exon embedding SRE region, which is a definition of splicing decision model that computationally predict the loss-of-function of SRE by SNP. Using the splicing decision model, we identified SNPs within SREs associated with exon skipping events.

### Functional annotation of differentially expressed exons

The impact of exon skipping events on protein structure and function was evaluated in silico. The skipped exons are translated into a functional domain, lead to out of frame through a frameshift, and change their corresponding protein structure using UniProt web browser and RaptorX [36].

### Neuroimaging and genotyping analysis

[^18^F] Florbetapir PET scans downloaded from the Alzheimer’s Disease Neuroimaging Initiative (ADNI) were pre-processed as described [37]. [^18^F] Florbetapir PET scans were intensity normalized by the whole cerebellum. The normalization yielded standardized uptake value ratio (SUVR) images. The ongoing ADNI study was launched in 2003 to test whether serial magnetic resonance imaging (MRI), PET, other biological markers, and clinical and neuropsychological assessment could be combined to measure the progression of MCI (mild cognitive impairment) and early AD, as published previously [38, 39] and found at www.adni-info.org. Genotyping for ADNI was performed using three different Illumina genotyping platforms. We imputed un-genotyped SNPs separately in each platform using MACH and the HRC (Haplotype Reference Consortium) data as a reference panel after standard sample and SNP quality control procedures and selection of only non-Hispanic Caucasian participants [40].

### Association of SNPs affecting exon skipping with AD-related neuroimaging endophenotype

First, an association analysis was performed for SNPs that potentially affect identified exon skipping events with a global cortical measure of amyloid-β deposition as measured by PET scans in an AD-related quantitative phenotype. The global cortical amyloid-β level was calculated as a mean regional SUVR value extracted for the frontal, parietal, temporal, limbic, and occipital lobes using the MarsBaR toolbox implemented in the Statistical Parametric Mapping 8 (SPM8) software (http://www.fil.ion.ucl.ac.uk/spm/software/spm8/) [41]. Also, a detailed whole brain-based neuroimaging analysis was performed using multivariate models for amyloid-β levels on voxel-by-voxel bases. Age at baseline, sex, and years of education were used as covariates for the association test. Correction for multiple comparisons was performed using false discovery rate (FDR) correction method at a 0.05 level of significance.

## Results

This study analyzed RNA-Seq data of hippocampus brain tissues from 74 participants from the ACT study. Participants were diagnosed as cognitively normal elder controls (CN) or AD patients. As shown in Table 1, there were 24 AD and 50 CN participants, and the mean Braak stages of AD and CN were 4.4 and 2.78, respectively.

**Table 1:** Demographic characteristics of 74 study samples

	# of sample	Male/Female	Age [min-max]	Education [min-max]	Braak stage
AD	24	11/13	91 (7.2) [78–100+]	14.4 (3.25) [6–21]	4.4 (1.70) [0–6]
CN	50	30/20	89 (6.9) [78–100+]	14.7 (3.07) [8–21]	2.76 (1.49) [0–6]

### Differentially expressed exons in AD hippocampus tissue

**Fig. 2:**
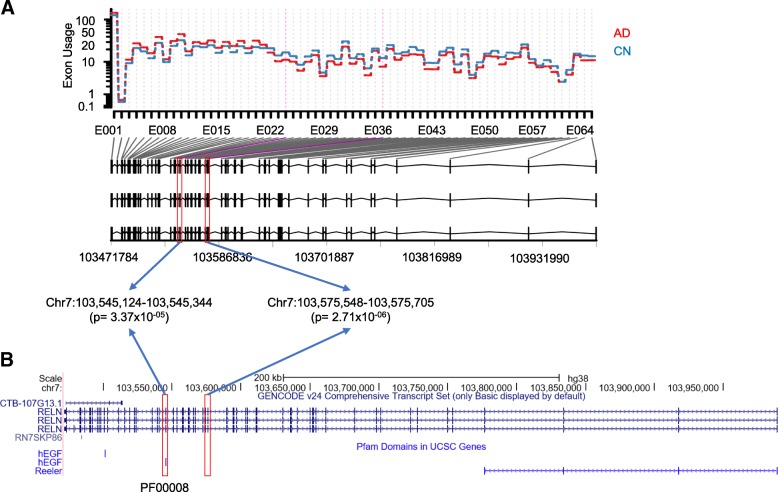
Two AD-associated exon skipping events in *RELN*. **a** Comparison of the expression levels of each exon between AD and CN participants, indicating two significant exon skipping events (exons 24 and 37) in the AD. **b** Exon structure and the hEGF domain encoded by the skipped exons (UCSC genome browser)

**Fig. 3:**
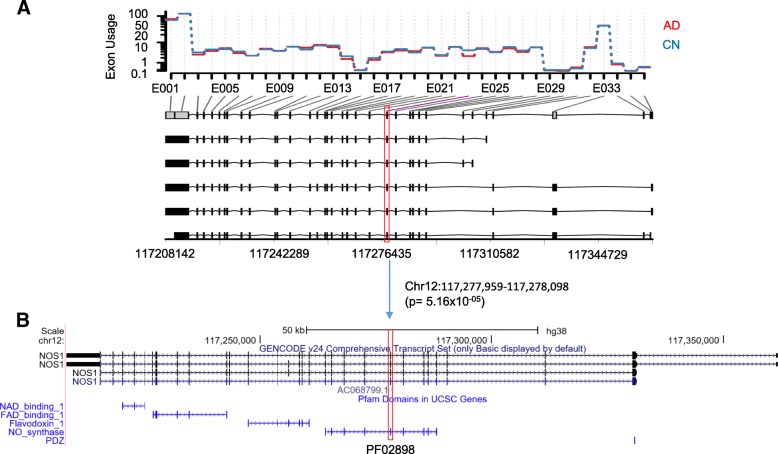
One AD-associated exon skipping event in *NOS1*. **a** Comparison of the expression levels of each exon between AD and CN participants, indicating a significant exon skipping event (exon 23) in the AD. **b** Exon structure and the NO_synthase domain encoded by the skipped exon (UCSC genome browser)

Using the computational pipeline described in Fig. 1, normalized expression levels were calculated for each exon in a genome-wide manner, and a generalized linear regression model was used to identify AD-associated exon skipping events. After adjusting for multiple comparisons using the FDR method, we identified three exon skipping events in two genes, *RELN* and *NOS1*, as significantly associated with the AD (FDR-corrected *p*-value < 0.05 and fold change > 1.5). Two exons in *RELN*, exons 24 and 37, showed significantly lower expression levels in AD patients compared to CN participants, suggesting that the exons tend to be skipped more in the AD (Fig. 2a). The exon 24 was predicted to encode a hEGF domain region (PF00008, Fig. 2b). Furthermore, for AD participants, we investigated whether the Braak stage is associated with the exon skipping event. In 24 AD participants, 19 were in the Braak stages 4, 5 and 6. Among the 19 AD participants in higher Braak stages, 15 showed significantly lower expression levels of the exon 24 compared to the other 4 AD participants (one-way chi-squared test, *p*-value = 0.01), suggesting that the exon tends to be skipped more in higher Braak stages of AD participants. In *NOS1*, exon 23 had lower expression levels in AD patients compared to CN participants (Fig. 3a) and was a part of the NO_synthase domain (PF02898, Fig. 3b). Our results suggest that these exon skipping events may affect the function of the corresponding protein products.

### Prediction of the effect of the identified exon skipping events on protein

**Fig. 4:**
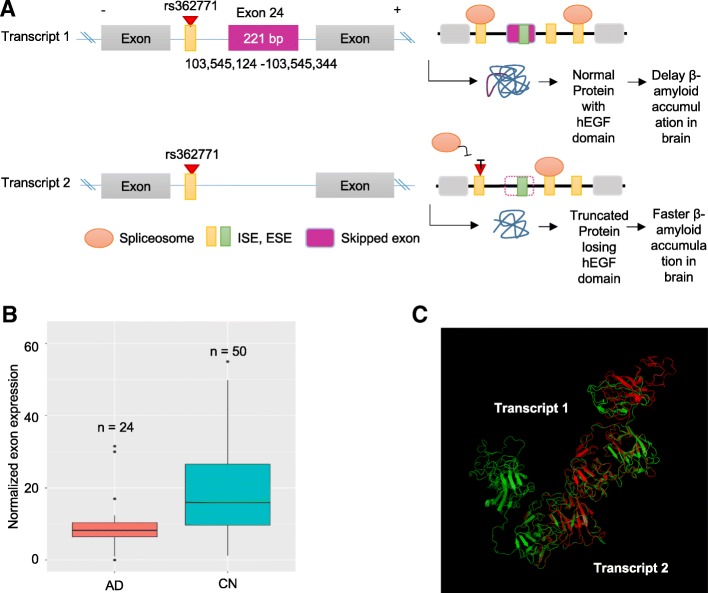
Functional impact of the AD-associated exon skipping event (exon 24) in *RELN*. **a** Schema of the potential functional implication of exon skipping and splicing-associated SNP. **b** Normalized expression levels for exon 24 between AD and CN participants. **c** Structure alignment of the pair of transcript1 retaining exon 24 (green) and transcript 2 with the exon skipping (red)

**Fig. 5:**
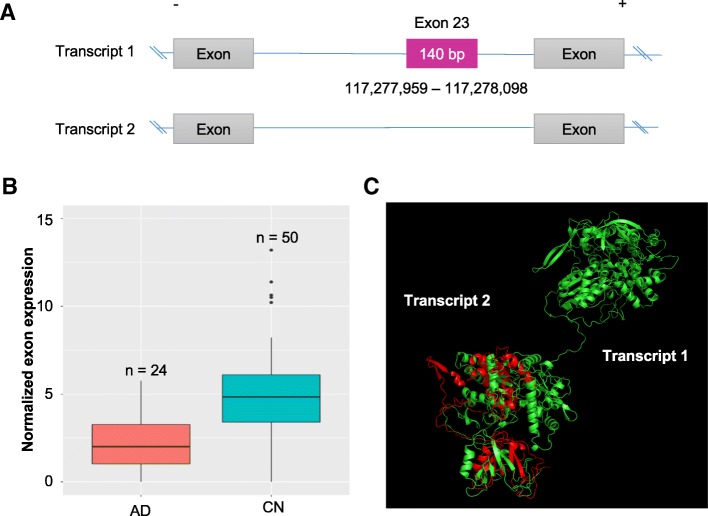
Functional impact of the AD-associated exon skipping event (exon 23) in *NOS1*. **a** Skipping of exon 23. **b** Normalized expression levels for exon 23 between AD and CN participants. **c** Structure alignment of the pair of transcript1 retaining exon 23 (green) and transcript 2 with the exon skipping (red)

To characterize the potential impact of the variants on the protein, each variant was analyzed using UniProt web browser and RaptorX. All of three skipped exons in *RELN* and *NOS1* resulted in producing a coding sequence which is out-of-frame, potentially generating an undesired protein product. As presented in Fig. 4, the first AD-associated exon skipping (exon 24) in *RELN* showed lower expression levels of the exon in AD compared to CN participants (Fig. 4b; FDR-corrected *p*-value = 0.034, fold change = 1.51). The adjacent exon 23 and 25 showed similar levels of difference but not significant (FDR = 0.154 for both exons, fold change = 1.39 and 1.37, respectively). The transcript (transcript1) retaining the exon 24, which encodes the hEGF, likely results in a functional version of the *RELN* gene, while the transcript which lacks exon 24 will lose the hEGF domain and thus produce the truncated protein product due to the out-of-frame of the exon length (Fig. 4a). Structural analysis of the truncated version of the protein suggests that exon skipping may be implicated in functional changes of *RELN* in the AD (Fig. 4c). The other exon (exon 37) in *RELN* is presented in Additional file 1: Figure S1. Additionally, exon 23 was identified as being skipped in *NOS1* (Fig. 5a). AD participants had significantly lower expression levels of the exon compared to CN (Fig. 5b; FDR corrected *p*-value = 0.043, fold change = 1.90). The transcript (transcript1) retaining the exon 23, which encodes a part of the NO_synthase, can be translated into the protein with normal functions of *NOS1*. In contrast, the transcript with the skipping of exon 23 may not only lose the NO_synthase domain but also produce the truncated protein due to the out-of-frame of the exon length. We also showed the partial loss of the protein structure due to the skipped exon using the protein 3D structure analysis (Fig. 5c).

### Association of SNPs affecting exon skipping with AD-related neuroimaging phenotypes

**Fig. 6:**
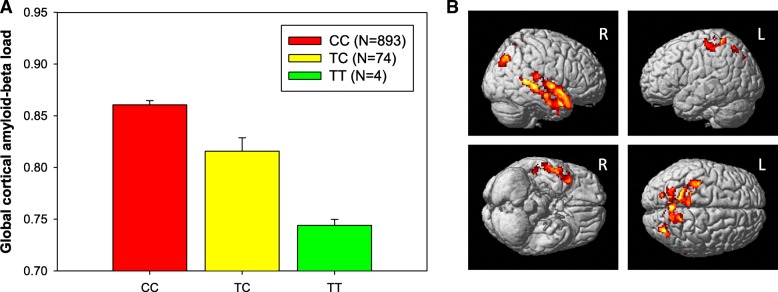
Regional effects (**a**; global cortical amyloid-β load) and voxel-wide association (**b**) of rs362771 in *RELN* affecting exon skipping with amyloid-β deposition

Next, we performed an association analysis of SNPs affecting exon skipping events with a global cortical measure of amyloid-β deposition as an AD-related quantitative phenotype. Using the splicing decision model, we first identified 46 and 11 SNPs in *RELN* and *NOS1*, respectively, potentially affecting the identified three exon skipping events (MAF > 1%) from HRC-based imputed ADNI GWAS data. We identified one SNP (rs362771) in intron adjacent to the skipped exon 24 in *RELN* as significantly associated with cortical amyloid-β levels (Fig. 6a; permutation-based corrected *p*-value < 0.05). Furthermore, we performed an unbiased whole-brain-based imaging association analysis using age, sex, years of education as covariates to assess the effect of rs362771 on whole-brain amyloid-β deposition and identified significant associations after adjustment for multiple comparisons using cluster-wide FDR procedure. The minor allele of rs362771 conferred decreases in cortical amyloid-β levels in the right temporal and bilateral parietal lobes (Fig. 6b). As shown in Fig. 4a, we found that the SNP (rs362771) is located within the ISE site (5th site of hexametric sequence *CCTTCC*), suggesting that the SNP may affect the exon skipping and the skipped exon may be associated with AD pathogenesis.

## Discussion

Here we developed a computational pipeline for the identification of exon skipping and a splicing decision model for the identification of SNPs affecting exon skipping. Altered expression patterns (exon skipping events) within two distinct exon regions of *RELN and NOS1* in the human hippocampus affected by Alzheimer’s disease (AD) were identified. Interestingly, expression levels of identified alternatively spliced exons are decreased in the AD and the minor allele of one SNP in *RELN* potentially affecting exon skipping negatively correlate with a global cortical amyloid-β burden. Our results indicate that essential exon regions for the *RELN and NOS1* genes are alternatively spliced in the AD hippocampus compared to cognitively normal elderly controls. It was previously reported that *RELN* delays amyloid-β fibril formation and rescues cognitive deficits in an AD model [42]. Two major neuropathological hallmarks of the AD are the accumulation of toxic levels of amyloid-β molecules and hyper-phosphorylated tau protein that leads to neurofibrillary tangles. Although it was known that *RELN* and *NOS1* were associated with neurologically related traits, the methodology presented here suggests new possible mechanisms by which they may influence AD pathology [43–45].

*RELN* is an extracellular matrix glycoprotein that plays a number of important roles in the central nervous system (CNS) and its dysfunction is associated with AD [46–48]. Many studies have found associations between genetic variation in *RELN* and neurological traits, often using case-control study design [49]. Characterization of these genetic associations is important however, it lacks mechanistic insight that can be provided with interrogating specific regions of the gene, or incorporating other molecular information, such as gene expression. By utilizing a neuroimaging endophenotype, along with transcriptomic information in relation to alternative splicing, we identified differential expression levels among exons in *RELN*. The function of the *RELN* protein is to help mediate cell migration during brain development by activating a signaling pathway through binding of cell surface proteins [46, 50]. These pathways mediate the process of tau phosphorylation and a reduction of *RELN* expression can significantly accelerate amyloid-β deposition in transgenic AD mice [48]. Thus, differential exonic usage with respect to the AD may produce an isoform of *RELN* protein that potentially accelerates amyloid-β deposition and/or mis-regulation of tau phosphorylation, both leading to AD-related phenotypes. Specific exonic expression appears to play an important role. Thus, understanding the role of exon skipping may lead to more consistent conclusions with respect to the role of *RELN* and other proteins [46].

*NOS1*, the other gene identified in this work, has also been of significant interest in relation to heart and neuronal function since nitric oxide (NO) is an important signaling molecule that is produced by NO-producing enzymes, such as NOS1 [51]. NOS can produce significant amounts of reactive oxygen species (ROS) that can lead to damaged proteins. Therefore, mis-splicing of *NOS1* could lead to increased ROS, and thus increased protein mis-folding and aggregation associated with neurodegenerative diseases (i.e. AD). Both increased and decreased expression of *NOS1* have been associated with a cognitive disruption in relation to the AD [52]. Therefore, it will be interesting to characterize how the expression of different isoforms and exons contributes to the spectrum of phenotypes related to AD in future work.

Although both genes had previous connections with neuronal development and splicing [46, 51], this study makes a novel association between alternative splicing and AD with respect to *NOS1* and *RELN*. Since both *RELN* and *NOS1* could exacerbate different mechanisms by which AD arises, it would be interesting for future work to investigate the co-occurrence of variants in these genes and how it impacts neurological function. While many advantages of the methodology employed here have been described in detail, it is also valuable to point out some limitations to avoid over-interpretation and reflect on what improvements need to be addressed in future work.

For instance, our sample size was moderate for this study with respect to the expression analysis. Thus, we may have missed some genes with alternative splicing in SRE SNPs associated with AD due to limited detection power. Previous work has suggested sex differences in AD [47]. The other limitation by the original dataset we used for exon expression quantification is that junction reads were excluded in the downloaded aligned bam file. As junction reads are important resources for estimating more accurate exon skipping events, inclusion of the junction reads may improve the power of our splicing decision model to detect differentially expressed exons in AD. Additionally, it is necessary for follow-up studies to test the function of exon skipping events experimentally as well as to investigate the epigenetic changes influencing the splicing decision – especially association of DNA methylation status in intragenic regions (exons and introns) [53–55]. Finally, here we only explored AD, but the methods applied here could be employed to understand the complex gene-trait relationship among other neurological diseases. In summary, through our integrative analysis of RNA-Seq, genomics, and neuroimaging data, the *RELN* and *NOS1* genes were identified as having differential exon usages with respect to the AD. This work also suggests applying an imaging genetics approach, along with utilizing SRE variants, will help shed light on previously unidentified gene-trait relationships.

Our study was not able to recapitulate a statistically significant exon skipping or spliced isoforms in any of the historical genes (i.e., including *APP* [13, 16], *PSEN1* [17], *PSEN2* [17], *APOE* [16], and *MAPT* [18–20]). However we observed there were the marginal signals of ten exons in those genes at the unadjusted *p*-value < 0.05 and fold changes between − 1.5 and 1.2, which might be due to the following reasons, 1) alternative splicing events occur in the tissue- and cell type- specific manner and are even more specific to brain regions [15, 16, 56, 57], 2) our study analyzed the RNA-Seq data from the hippocampus regions whereas the known alternative splicing events in the historical genes were found in the whole brain or cerebral cortex region, and 3) as mentioned above, we might miss a signal of those genes under the lack of detection power (AD = 24 and non-AD elderly controls = 50).

## Conclusion

In conclusion, this study provides evidence that our novel approach can identify significant exon skipping events and genetic variation potentially affecting exon skipping in Alzheimer’s disease. Between *RELN* and *NOS1,* three exons are altered in their expressions in the human hippocampus affected by Alzheimer’s disease. It also suggests that the functional relationship between exon skipping and protein structures for *RELN* and *NOS1* may be altered in AD pathology. Further studies are needed to better understand the functional role that identified exon skipping and SNPs affecting exon skipping play in AD pathophysiology. Integration of multiple omics and neuroimaging data in a systems biology approach will provide valuable insights into a possible mechanism underlying AD pathology through exon skipping, thus potentially helping identify novel therapeutic targets.

## Additional file

Additional file 1: **Figure S1.** Functional impact of the AD-associated exon skipping event (exon 37) in RELN. (A) Schema of the potential functional implication of exon skipping and splicing-associated SNP. (B) Normalized expression levels for exon 37 between AD and CN participants. (C) Structure alignment of the pair of transcript1 retaining exon 37 (green) and transcript 2 with the exon skipping (red). (PDF 58 kb)

## Abbreviations

AD: Alzheimer’s disease
CN: Control normal
ESEs: Exonic splicing enhancers
ESSs: Exonic splicing silencers
FDR: False discovery rate
hEGF: Human Epidermal Growth Factor
ISEs: Intronic splicing enhancers
ISSs: Intronic splicing silencers
PET: Florbetapir position emission tomography
SNP: Single nucleotide polymorphism
SRE: Splicing regulatory element
