# Supplementary material for: Identification of exon skipping events associated with Alzheimer’s disease in the human hippocampus
Source: BMC Med Genomics. 2019 Jan 31;12(Suppl 1):13. doi: 10.1186/s12920-018-0453-8 (PMC6357347; doi:10.1186/s12920-018-0453-8)
Supplement: Supplementary file 1 — Figure S1. Functional impact of the AD-associated exon skipping event (exon 37) in RELN. (A) Schema of the potential functional implication of exon skipping and splicing-associated SNP. (B) Normalized expression levels for exon 37 between AD and CN participants. (C) Structure alignment of the pair of transcript1 retaining exon 37 (green) and transcript 2 with the exon skipping (red). (PDF 58 kb) [file 12920_2018_453_MOESM1_ESM.pdf]

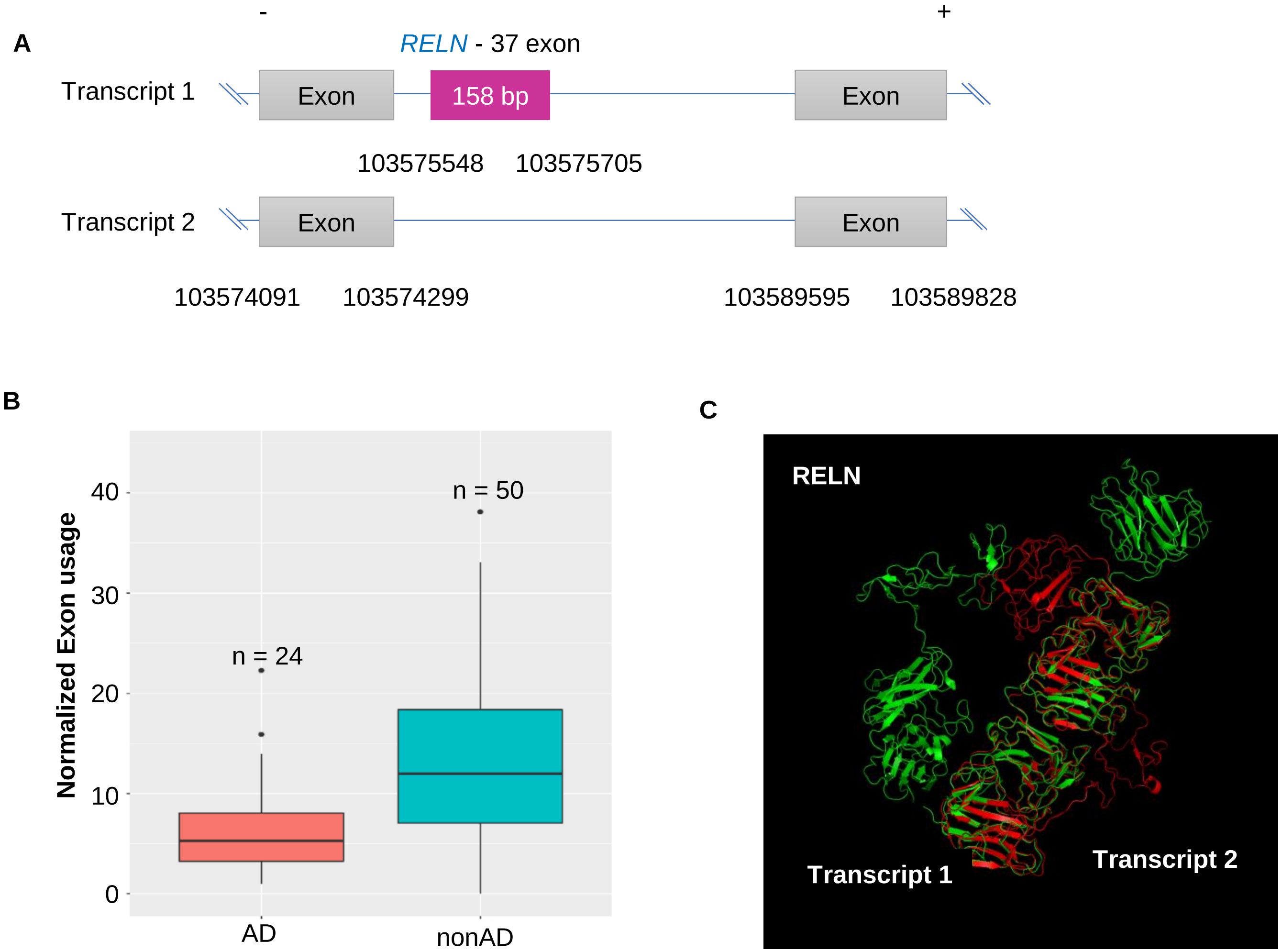

Figure S1. Functional impact of the AD-associated exon skipping event (exon 37) in *RELN*. (A) Schema of the potential functional implication of exon skipping and splicing-associated SNP. (B) Normalized expression levels for exon 37 between AD and CN participants. (C) Structure alignment of the pair of transcript1 retaining exon 37 (green) and transcript 2 with the exon skipping (red).
